# Combined Effects of *ESRα* DNA Methylation and Progesterone on Glucose Metabolic Disorders: The Henan Rural Cohort Study

**DOI:** 10.3390/nu15071659

**Published:** 2023-03-29

**Authors:** Bo Feng, Lulu Wang, Dandan Wei, Wenqian Huo, Tao Jing, Chongjian Wang, Zhenxing Mao

**Affiliations:** 1 Department of Epidemiology and Biostatistics, College of Public Health, Zhengzhou University, 100 Kexue Avenue, Zhengzhou 450001, China; 2 Department of Occupational and Environmental Health Sciences, College of Public Health, Zhengzhou University, Zhengzhou 450001, China; 3 School of Public Health, Tongji Medical College, Huazhong University of Science and Technology, Wuhan 430074, China

**Keywords:** type 2 diabetes mellitus, combined effect, estrogen receptor alpha, DNA methylation progesterone, impaired fasting glucose

## Abstract

To explore the independent and combined effects of *ESRα* methylation and progesterone on impaired fasting glucose (IFG) and type 2 diabetes mellitus (T2DM), a case-control study including 901 subjects was conducted. Generalized linear models were performed to assess the independent and combined effects of *ESRα* methylation and progesterone on IFG or T2DM. Methylation level of cytosine-phosphoguanine (CpG) 1 in the estrogen receptor α (*ESRα*) gene was positively related to IFG in both men (odds ratio (*OR*) (95% confidence interval (*CI*)): 1.77 (1.05, 3.00)) and postmenopausal women (*OR* (95% *CI*): 1.82 (1.09, 3.04)), whereas the association between CpG 1 and T2DM was not significant. Positive associations of progesterone with IFG and T2DM were observed in both men (*OR* (95% *CI*): 2.03 (1.18, 3.49) and 3.00 (1.63, 5.52)) and postmenopausal women (*OR* (95% *CI*): 2.13 (1.27, 3.56) and 3.30 (1.85, 5.90)). Participants with high CpG 1 methylation plus high progesterone had an increased risk of IFG and T2DM, both in men and postmenopausal women. *ESRα* methylation and progesterone were positively associated with IFG, and the positive association between progesterone and T2DM was also found. Importantly, we firstly found the combined effects of *ESRα* methylation and progesterone on IFG and T2DM.

## 1. Introduction

Diabetes, as a serious, chronic condition, has placed a heavy economic burden on society. Updated data show that there were approximately 537 million adults worldwide with diabetes, of which up to 140.9 million were in China, ranking first around the world [1]. A survey conducted in rural China estimated that the age-standardized prevalence of type 2 diabetes mellitus (T2DM) in rural areas had reached 6.98% [2]. Impaired fasting glucose (IFG) is a type of prediabetes, defined as the condition of raised blood glucose levels above the normal range and below the diabetes diagnostic threshold [1]. Patients with IFG have a substantially increased risk of developing diabetes [3,4,5,6]. A recent study proposed that the prevalence of IFG, even among the undiagnosed Chinese rural population, was 7.22% [7]. Implementing preventive interventions can delay or halt the progression from IFG to diabetes [8]. Given these, it is critical to identify risk factors of IFG and T2DM to take effective interventions.

Mounting studies have noted that epigenetic mechanisms might be involved in the formation and progression of aberrant glucose metabolism, in particular DNA methylation [9,10,11]. Several studies in humans and rodents models suggested that, at physiological levels, oestradiol was involved in maintaining normal glucose homeostasis [12,13]. Additionally, the beneficial metabolic effects of estrogens are mainly mediated by estrogen receptor α (ESRα), such as anti-lipogenesis and improvement of insulin sensitivity and glucose tolerance [14,15,16]. Animal experiments have found that *ESRα* knockout mice demonstrated insulin resistance (IR), glucose intolerance, and obesity [17,18,19,20]. Furthermore, the man with an inactivating mutation of the *ESRα* gene was reported to exhibit IR, glucose intolerance, and hyperinsulinemia [21]. The function of ESRα could be silenced by *ESRα* promoter hypermethylation [22,23,24]. Meanwhile, *ESRα* methylation has been shown to increase the risk of many diseases, such as breast cancer [25], hepatocellular carcinoma [26], and colorectal cancer [27]. However, little research has explored the association between *ESRα* methylation and disturbed glucose metabolism.

Progesterone is an endogenous hormone essential for reproduction [28], neurological function [29], and immune regulation [30]. Many experimental data have linked elevated progesterone to glucose disturbance [31,32,33]. As for population studies, a cross-sectional study conducted in Germany found positive associations of progesterone with fasting glucose and HbA1c in postmenopausal women [34]. Recently, a retrospective study has reported that patients with 17-hydroxylase/17,20-lyase deficiency were susceptible to abnormal glucose metabolism due to high levels of progesterone [35]. Furthermore, progesterone could contribute to IR by suppressing the phosphatidylinositol-3-kinase (PI3K)/Akt pathway [36]. Conversely, ESRα mediated the effect of estrogen to increase protein kinase B (AKT) phosphorylation and glucose transporter 4 (GLUT4) expression, consequently improving insulin sensitivity [37,38,39,40]. Moreover, there was growing evidence for cross-talk between ESR and progesterone receptor pathways [41,42]. Meanwhile, we have previously found that prediabetes and T2DM were positively linked to progesterone in a rural Chinese population [43]. Therefore, we hypothesized that there might be combined effects of *ESRα* methylation and progesterone on T2DM or IFG. However, few studies examined this.

Given these, we designed this case-control study to assess the independent associations of *ESRα* methylation in cytosine-phosphoguanine (CpG) 1 and progesterone with IFG and T2DM, and then we explored their combined effects on IFG and T2DM among the Chinese rural population.

## 2. Materials and Methods

### 2.1. Study Design and Population

The study subjects were selected from the Henan Rural Cohort (registration number: ChiCTR-OOC-15006699), a prospective population-based cohort study of chronic non-communicable diseases. The baseline survey was conducted from July 2015 to September 2017 in Suiping, Yuzhou, Tongxu, Yima, and Xinxiang counties of Henan Province, China, with a total of 39,259 subjects recruited through a multi-stage stratified whole-group sampling. Detailed information about the cohort study has been previously described [44].

In this case-control study, we first included 925 T2DM patients aged 18 to 79, and 925 IFG patients and 925 controls were matched to the T2DM patients based on being the same age (±3 years) and gender. Due to the massive impact of the menstrual cycle on progesterone levels, premenopausal women were excluded (*n* = 301). Participants lacking data on *ESRα* gene methylation levels (*n* = 1536) or progesterone levels (*n* = 5) were also excluded. Participants missing information on homeostasis model assessment (HOMA)2-β (*n* = 1), insulin (INS) (*n* = 17), or glycosylated hemoglobin A1c (HbA1c) (*n* = 14) were further excluded. The study ultimately included 901 subjects, including 338 normal glucose tolerance (NGT), 249 IFG patients, and 314 T2DM. All participants signed a written informed consent prior to inclusion in the study.

The study was conducted in accordance with the guidelines of the Declaration of Helsinki and was approved by the Ethics Committee of the Zhengzhou University Life Science (code: [2015] MEC (S128)).

### 2.2. Data Collection

Basic characteristics of participants regarding socio-demographic characteristics (including gender, age, marital status, education level, per capita monthly income, and family history of diabetes) and lifestyles (including smoking status, drinking status, and physical activity) were collected by trained enumerators using a structured questionnaire. Detailed criteria for the grouping of these variables have been reported previously [45]. In brief, participants who had at least one parent or sibling diagnosed with T2DM were defined as those with a family history of T2DM. More than 12 months without a menstrual period was defined as menopause in female participants.

Anthropometric measurements, such as height (m) and weight (kg), were taken by trained researchers [45]. Body mass index (BMI) was calculated as weight divided by squared height (kg/m^2^). The blood pressure was measured using a sphygmomanometer (Omron HEM-7071A) in triplicate at 30-s intervals in the sitting position. HOMA2-IR and HOMA2-β were calculated by the updated computer-based homeostasis model introduced by Wallace [46,47].

### 2.3. Laboratory Measurements

Venous blood samples were drawn from subjects after fasting for at least 8 h, centrifuged on site, and stored in a −80 °C refrigerator. Lipid levels, including total cholesterol (TC), triglyceride (TG), high-density lipoprotein cholesterol (HDL-C), and low-density lipoprotein cholesterol (LDL-C), were measured by enzymatic methods using the ROCHE Cobas C501 automatic biochemical analyzer; fasting plasma glucose (FPG) was measured by the glucose oxidase method. HbA1c was measured by high performance liquid chromatography (HPLC) using the Bio-Rad VARIANT II analyzer. INS was detected by radioimmunoassay.

The serum progesterone levels were determined using liquid chromatography-tandem mass spectrometry (LC-MS/MS) (a Waters XEVO TQ-S system (Waters, Milford, MA, USA)). To reduce measurement bias, blind determination was employed, and a blank and a quality control sample were detected after every 12 samples tested [48].

### 2.4. Methylation Analysis

Genomic DNA was extracted from whole blood samples using the Whole Blood Genomic DNA Extraction Kit III (Magnetic bead) (Bioteke Corporation, Beijing, China). Three CpG islands (Length 231 (152128479-152128709), Length 237 (152129066-152128830) and Length 233 (152129651-152129883)), including 49 sites located in the *ESRα* promoter, were sequenced and detected their methylation levels using MethylTarget™ (Genesky Corporation, Shanghai, China) following bisulfite sequencing [49,50]. Information on the measurement, primer sequences, genomic regions, and CpG sites are summarized (Supplementary Tables S1 and S2).

### 2.5. Ascertainment of Cases

According to the diagnostic criteria recommended by the American Diabetes Association (ADA) (2002) and the World Health Organization (WHO) (1999) guidelines, after excluding type 1 diabetes, gestational diabetes, and other specific types of diabetes, IFG was defined as subjects meeting one of the following conditions: (1) 6.1 mmol/L ≤ FPG < 7.0 mmol/L; (2) 5.7% ≤ HbA1C < 6.5%. T2DM was defined as subjects meeting one of the following conditions: (1) FPG ≥ 7.0 mmol/L; (2) HbA1c ≥ 6.5%; (3) diagnosed with T2DM by physicians and taking anti-glycemic drugs during the previous two weeks.

### 2.6. Statistical Analysis

The basic characteristics were presented as mean ± (standard deviations (SD)) (normal distribution) or median (interquartile ranges (IQR)) (skewed distribution) for continuous variables and numbers (percentages) for categorical variables. The differences in the distribution of normal continuous, skewed continuous, and categorized variables between the case and control groups were compared using *t*-tests, Mann-Whitney U tests, and Chi-square tests, respectively.

The associations of *ESRα* methylation with IFG or T2DM were examined with logistic regression model. Two-sided *p* < 0.05/49 indicated significance when exploring the association between each CpG site and IFG or T2DM. Then, we explored the relationships of 3 CpG regions with IFG and T2DM, based on which a significant association was found between CpG 1 (Chr6: 152128479_Chr6: 152128709) and IFG. Moreover, to investigate the associations of *ESRα* methylation and progesterone with different glucose statuses, *ESRα* methylation (CpG 1) and progesterone were divided into dichotomous variables by their corresponding median values. Sex-stratified analysis was conducted throughout the research due to sex differences in serum progesterone levels. Additionally, to estimate potential non-linear relationships, levels of CpG 1 methylation and progesterone were categorized in tertiles (T), with the lowest tertile group (T1) defined as the reference. Generalized linear models were utilized to examine the effects of CpG 1 methylation and progesterone on IFG and T2DM, with the effect estimates expressed as odds ratios (*OR*s) and 95% confidence intervals (*CI*s) for IFG and T2DM by tertiles and dichotomies of variables. Furthermore, to study the dose–response relationships, trend tests were conducted by entering tertiles as continuous variables.

Two models were constructed in the analysis: the crude model and the adjusted model (adjusted for BMI, smoking status, drinking status, physical activity, per capita monthly income, level of education, family history of T2DM, SBP, PP, TC, TG, HDL-C, and LDL-C).

HOMA2-β was natural logarithm-transformed into Ln-HOMA2-β due to its skewed distributions. After adjusting multiple variables, linear regression was utilized to estimate the relationships between progesterone, CpG 1 methylation, and glucose homeostasis markers (including FPG, HbA1c, INS, HOMA2-IR, and Ln-HOMA2-β).

Furthermore, to examine the combined effects of CpG 1 methylation level and progesterone on IFG and T2DM, the terms of the corresponding dichotomies were included in logistic regression model.

Finally, considering the potential effects of alcohol intake and smoking on DNA methylation and disturbance of glucose metabolism [51,52,53], stratified analyses by alcohol intake (drinking now and no drinking now) and smoking status (smoking now and no smoking now) were examined in the adjusted model among men, since most postmenopausal female subjects were non-drinkers and non-smokers.

All analyses were performed in SPSS software (version 21.0), and R Language software (version 4.2.2), and a two-tailed *p* < 0.05 was considered statistically significant.

## 3. Results

### 3.1. Basic Characteristics

**Figure 1:**
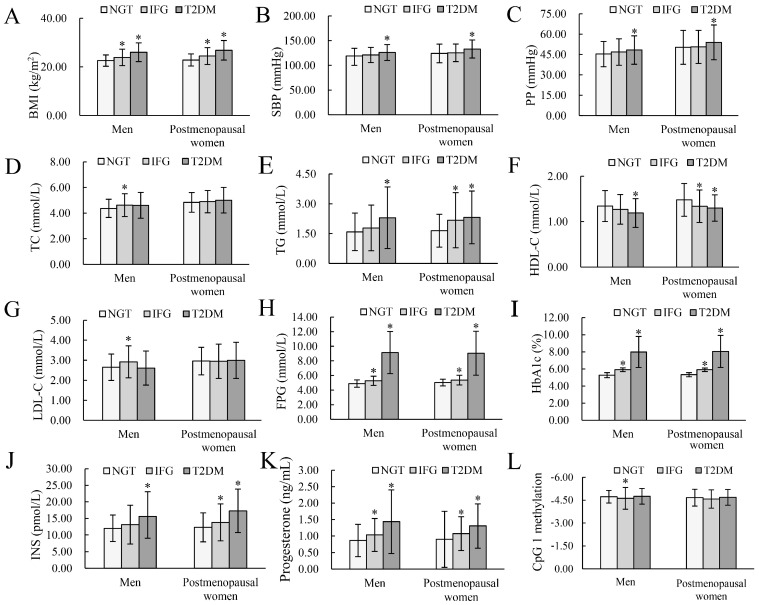
Basic characteristics of the study population by gender. (**A**–**L**) display the distribution of the indices BMI, SBP, PP, TC, TG, HDL-C, LDL-C, FPG, HbA1c, INS, progesterone, CpG 1 methylation in men and postmenopausal women, respectively. BMI, body mass index; CpG, cytosine-phosphoguanine; FPG, fasting plasma glucose; HDL-C, high-density lipoprotein cholesterol; HbA1c, glycosylated hemoglobin A1c; INS, insulin; IFG, impaired fasting glucose; LDL-C, low-density lipoprotein cholesterol; NGT, normal glucose tolerance; PP, pulse pressure; SBP, systolic blood pressure; TC, total cholesterol; TG, triglyceride; T2DM, type 2 diabetes mellitus. * Compared with NGT, *p* < 0.05.

The basic characteristics of participants are shown in Table 1 and Figure 1. In men, compared to NGT, patients with IFG were more likely to have higher levels of BMI, TC, LDL-C, FPG, and HbA1c; T2DM patients were more likely to be smokers and to have higher BMI, proportions of family history of T2DM, SBP, PP, TG, FPG, HbA1c, and INS, as well as lower HDL-C. In postmenopausal women, individuals with IFG and T2DM seem to have higher levels of BMI, TG, FPG, HbA1c, and INS, as well as lower HDL-C than those with NGT. Additionally, T2DM patients who were postmenopausal women had higher levels of SBP, PP, and TG, as well as higher proportions of family history of T2DM.

Compared with NGT, participants with IFG and T2DM had higher progesterone in both men and postmenopausal women, whereas only IFG men tended to be with higher CpG 1 methylation level (all *p* < 0.05).

### 3.2. Independent Effects of ESRα Methylation and Progesterone on IFG and T2DM

As Supplementary Table S3 shown, the positive associations between *ESRα* methylation sites (including *Chr6: 152128537*, *Chr6: 152128584*, *Chr6: 152128631*, and *Chr6: 152129681*) and IFG were found. The associations of 3 CpG islands with IFG and T2DM were summarized in Supplementary Table S4. The positive correlation was only found between CpG 1 and IFG among men and postmenopausal women.

Table 2 presents the associations of CpG 1 methylation and progesterone with IFG and T2DM in tertiles and dichotomies. After adjusting for confounders, a positive relationship was found between CpG 1 methylation and IFG both in men (*OR* (95% *CI*): 1.77 (1.05, 3.00)) and postmenopausal women (*OR* (95% *CI*): 1.82 (1.09, 3.04)), whereas the association between CpG 1 and T2DM was not significant. Additionally, when CpG 1 methylation level was analyzed as tertiles, compared with the T1, only the T3 was correlated with a 102% (*OR* (95% *CI*): 2.02 (1.06, 3.84)) and 98% (*OR* (95% *CI*): 1.98 (1.06, 3.72)) higher risk of IFG in men and postmenopausal women. Additionally, the effect became stronger as tertiles increased (*P*-trend < 0.05).

Likewise, progesterone was positively correlated with the prevalence of IFG and T2DM among men (*OR* (95% *CI*): 2.03 (1.18, 3.49) and 3.00 (1.63, 5.52)) and postmenopausal women (*OR* (95% *CI*): 2.13 (1.27, 3.56) and 3.30 (1.85, 5.90)) in the adjusted model. Likewise, the T3 of progesterone was significantly related to a higher risk of IFG and T2DM versus T1 (men: *OR* (95% *CI*): 2.26 (1.15, 4.47) and 6.40 (2.83, 14.45), including for postmenopausal women—*OR* (95% *CI*): 2.65 (1.42, 4.95) and 5.28 (2.52, 11.08)). The effect was heightened by the increasing tertiles (*P*-trend < 0.05).

In short, methylation level of CpG 1 of *ESRα* and progesterone were positively associated with IFG, and the positive association between progesterone and T2DM was also found.

### 3.3. Associations of ESRα Methylation (CpG 1) and Progesterone with Glucose Homeostasis Markers

**Figure 2:**
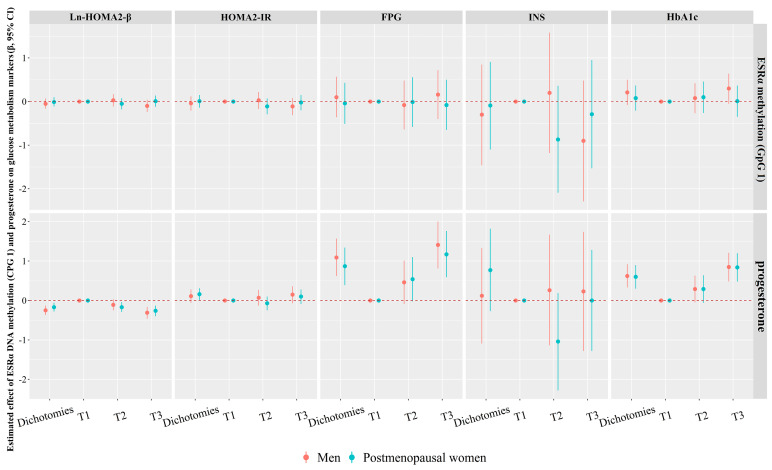
Associations of *ESRα* methylation (CpG 1) and progesterone with glucose homeostasis markers in men and postmenopausal women. Adjusted for BMI, smoking status, alcohol intake, physical activity, per capita monthly income, level of education, family history of T2DM, SBP, PP, TC, TG, HDL-C, and LDL-C; *CI*, confidence interval; CpG, cytosine-phosphoguanine; *ESRα*, estrogen receptors α; FPG, fasting plasma glucose; HOMA: homeostasis model assessment; HbA1c, glycosylated hemoglobin A1c; IR: insulin resistance; INS, insulin; Ln-, natural log-transformed; T, tertiles.

The relationships of CpG 1 methylation of *ESRα* and progesterone with glucose homeostasis markers are summarized in Figure 2. After multivariate adjustment, participants experiencing high progesterone levels were related to a 11% (95% *CI*: −6%, 28%) and 16% (95% *CI*: 1%, 31%) higher HOMA2-IR, a 1.09 mmol/L (95% *CI*: 0.62, 1.57) and 0.87 mmol/L (95% *CI*: 0.39, 1.34) higher FPG, a 0.62 mmol/L (95% *CI*: 0.33, 0.92) and 0.60 mmol/L (95% *CI*: 0.30, 0.90) higher HbA1C, and a 25% (95% *CI*: −37%, −13%) and 17% (95% *CI*: −28%, −6%) lower Ln-HOMA2-β compared with those who experienced low progesterone levels in men and postmenopausal women, respectively. Compared with T1, the T3 of progesterone was related to a 31% (95% *CI*: −46%, −16%) and 26% (95% *CI*: (−40%, −13%) decrease in Ln-HOMA2-β, an 1.41mmol/L (95% *CI*: 0.81, 2.00) and 1.71 mmol/L (95% *CI*: 0.59, 1.76) increase in FPG, and an 0.85mmol/L (95% *CI*: 0.48, 1.21) and 0.84 mmol/L (95% *CI*: 0.48, 1.20) increase in HbA1c in men and postmenopausal women. However, the relationships between CpG 1 methylation and glucose homeostasis markers were not significant.

Progesterone was positively correlated with HOMA2-IR, FPG, and HbA1C, whereas it was inversely correlated with Ln-HOMA2-β in both men and postmenopausal women.

### 3.4. Combined Effects of ESRα Methylation (CpG 1) and Progesterone on IFG and T2DM

**Figure 3:**
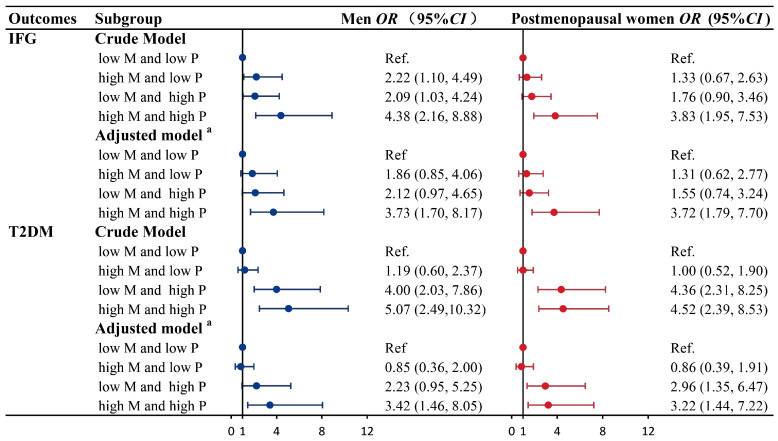
Combined effects of *ESRα* methylation (CpG 1) and progesterone on IFG and T2DM in men and postmenopausal women. ^a^: adjusted for BMI, smoking status, alcohol intake, physical activity, per capita monthly income, level of education, family history of T2DM, SBP, PP, TC, TG, HDL-C, and LDL-C; *CI*, confidence interval; CpG, cytosine-phosphoguanine; *OR*, odds ratio; M, *ESRα* methylation; P, progesterone; Ref., reference, with (1, 1) as the reference.

Figure 3 presents the combined effect of CpG 1 methylation of *ESRα* and progesterone on IFG and T2DM. After adjusting for confounders, combined effects of CpG 1 methylation and progesterone on IFG and T2DM were observed both in men and postmenopausal women. Compared with participants with low levels of CpG 1 methylation and progesterone, the combination of high levels of CpG 1 methylation and progesterone is more susceptible to IFG (men: *OR* (95% *CI*): 3.73 (1.70, 8.17). The following values were obtained: postmenopausal women: *OR* (95% *CI*): 3.72 (1.79, 7.70)) and T2DM (men: *OR* (95% *CI*): 3.42 (1.46, 8.05); postmenopausal women: *OR* (95% *CI*): 3.22 (1.44, 7.22)).

### 3.5. Stratification Analysis

The results of the stratified analysis are shown in Supplementary Tables S5 and S6. When stratified by alcohol intake, the association of CpG 1 methylation of *ESRα* with IFG was only significant among men with no drinking now. Interestingly, a relationship between the T3 of CpG 1 methylation and T2DM emerged in the “No drinking now” group (T3 vs. T1: *OR* (95% *CI*): 2.55 (1.01, 6.48)). Progesterone was significantly positively related to IFG and T2DM only in the “No drinking now” group, and the association of tertiles of progesterone with T2DM appeared to be stronger (T3 vs. T1: *OR* (95% *CI*):12.27 (4.03, 37.39)). When stratified by smoking status, progesterone was significantly related to IFG and T2DM only in men not smoking now. The positive associations have not altered substantially. No significant associations of CpG 1 methylation with IFG and T2DM were observed across smoking status categories in men.

As shown in Supplementary Figure S1, the combined effects of *ESRα* methylation (CpG 1) and progesterone on IFG and T2DM were only found in men with no drinking now or no smoking now.

## 4. Discussion

This case-control study was conducted to explore the independent and combined effects of *ESRα* methylation and progesterone on different glucose statuses. We found a positive association of progesterone with IFG and T2DM and a positive association between CpG 1 methylation of *ESRα* gene and IFG. Furthermore, combined effects of CpG 1 methylation and progesterone on IFG and T2DM were observed in this study, implying that people exposed to high levels of CpG 1 methylation of *ESRα* gene and progesterone had a higher risk of developing glucose metabolic disorders compared to other subgroups.

The present study found that high *ESRα* methylation levels were correlated with high IFG risk. At present, direct studies on *ESRα* methylation are lacking, but some indirect evidence can provide support for the results of this study. A previous study conducted on Swedish men evidenced that *ESRα* gene polymorphism was associated with fasting plasma glucose [54]. Moreover, considerable experimental data have reported that ESRs, especially ESRα, perform key roles in glucose homeostasis, including the regulation of insulin sensitivity, adiposity, and pancreatic β-cell function [55], and *ESRα* knockout mice exhibited increased fasting blood glucose levels and IR [19,20]. Considering that *ESRα* methylation could decrease the expression level of *ESRα*, *ESRα* methylation might have effects on blood glucose metabolism. The corresponding explanation is that ESRα-mediated estradiol (E_2_) effect activates PI3K/AKT pathway by promoting nucleus-plasma membrane shuttle of ESRα, increasing AKT phosphorylation and increasing GLUT4 expression translocation, thereby improving insulin-stimulated glucose uptake [37,38]. ESRα has also been shown to contribute to the tyrosine phosphorylation of insulin receptor substrate-1 (IRS-1) protein, which occurs prior to PI3K/AKT activation [39,40]. In addition, ESRα has been reported to mediate the effect of E_2_ in protecting pancreatic β-cells from apoptosis induced by oxidative stress [56]. As known, DNA hypermethylation of the *ESRα* promoter region can lead to the downregulation of *ESRα* expression [22,23,24]. Disruption of ESRα function due to lower gene expression results in increased insulin insensitivity and apoptosis of pancreatic β-cells, which may lead to an increased risk of IFG.

Several studies have linked progesterone with abnormal glucose metabolism [31,33,34,35,57,58,59]. Consistent with our results, an eight-year longitudinal study of Swedish opposite-sex twins indicated that progesterone was associated with markers of insulin resistance [57]. Nevertheless, one subsequent longitudinal study failed to find an association between progesterone with diabetes and insulin metabolism [58]. A cross-sectional study conducted in China reported that progesterone was significantly decreased in T2DM and impaired glucose tolerance patients [59]. In our study, however, progesterone was found to be positively correlated with IFG and T2DM, as well as glucose homeostasis markers HOMA2-IR, FPG, and HbA1C, whereas it was inversely correlated with Ln-HOMA2-β. The differences in findings may stem from the ethnicity and sample size of the study population, different outcomes of interest, and other confounding factors. Biochemical evidence offers possible explanations for the association between progesterone and glucose disturbances. Progesterone contributed to IR by promoting IRS-1 degradation and by inhibiting GLUT4 translocation in the IRS/PI3K/AKT pathway [36]. Furthermore, the Cb1/TC10 pathway functioned in parallel with the PI3K pathway [60], and progesterone hindered TC10 activation by decreasing Cbl phosphorylation [36]. Additionally, progesterone acted at the plasma membrane to blockade the L-type voltage-dependent Ca^2+^ channels in rat β-cells, inhibiting insulin release [61]. Besides, progesterone can induce apoptosis of insulin-secreting cells by triggering endoplasmic reticulum stress [32]. In a nutshell, progesterone may promote the development of IFG and T2DM and affect glucose homeostasis markers by leading to increased IR and decreased glucose intake, reducing insulin release, and inducing apoptotic death of insulin-producing cells. However, the exact mechanism still needs to be further explored.

It has been reported that progesterone might downregulate *ESR* expression [62,63]. We further explored the combined associations of *ESRα* methylation and progesterone levels on different glucose statuses, finding that individuals with high levels of CpG 1 methylation of *ESRα* gene and progesterone exhibited a significantly higher prevalence of IFG and T2DM compared with individuals with low levels of CpG 1 methylation of *ESRα* gene and progesterone. The mechanism of the combined effect of *ESRα* methylation and progesterone in the pathology of glucose disorder remains unclear, but there are several reasonable explanations. In the PI3K/AKT pathway, progesterone promoted down-regulation of IRS-1 protein, upstream of the PI3K/AKT activation and suppressed the subsequent phosphorylation of Akt, and it inhibited GLUT4 translocation and glucose uptake in a step distal to Akt phosphorylation [36]. To the contrary, up-regulation of PI3K/Akt signaling was observed by ESRα-mediated E_2_ effect through enhanced activity of IRS-1, increased AKT phosphorylation, and increased insulin-stimulated GLUT4 translocation [37,38,39,40]. The high level of *ESRα* methylation led to low expression of its protein, resulting in reduced progesterone antagonism of ESRα on the common PI3K/AKT signaling pathway. Moreover, progesterone caused an augmentation of oxidative species generation in RINm5F insulin-producing cells, and low expression of *ESRα* impairs the ability to circulate E_2_ to prevent β-cell apoptosis after exposure to oxidative stress, thus the combination of high *ESRα* methylation and high progesterone may lead to a greater susceptibility to IFG and T2DM. However, the exact biological mechanisms need to be further investigated.

To our knowledge, this was the first study which has focused on combined effects of *ESRα* methylation and progesterone on T2DM and IFG. Nonetheless, several limitations in the study need to be considered. Firstly, this study is a case-control design with the possibility of reverse causality, and the findings of this study need to be further validated in a large prospective cohort study. Secondly, the *ESRα* methylation was determined from whole blood, thus the results might be perturbed by the different leukocyte subtypes. Thirdly, the methylation status of peripheral blood mononuclear cells may differ from that of other tissues. Ultimately, our participants were confined to Henan rural areas in China, so the conclusion may be limited to extended to other countries and areas.

## 5. Conclusions

In a word, progesterone levels were positively related to T2DM. Meanwhile, both methylation level of CpG 1 in the *ESRα* gene and progesterone were positively related to increased risk of IFG, and they have combined effects on IFG and T2DM, indicating that maintaining low levels of CpG 1 methylation of *ESRα* gene and progesterone could decrease the risk of glucose metabolic disorders. These findings provide preliminary clues to the combined effect of *ESRα* methylation and progesterone on glucose disturbance. However, prospective studies are warranted to verify these associations.

## Figures and Tables

**Table 1:** Basic characteristics of the study population by gender.

Variables	Men			Postmenopausal Women		
	NGT	IFG	T2DM	NGT	IFG	T2DM
Subjects, n	160	124	147	178	125	167
Age (years)	61.00 (54.00, 65.00)	61.00 (54.00, 66.00)	61.00 (54.00, 65.00)	63.00 (57.75, 66.00)	63.00 (58.00, 66.00)	63.00 (58.00, 66.00)
BMI (kg/m^2^)	22.23 (20.75, 24.26)	23.93 (3.39) *	25.86 (23.57, 29.15) *	22.56 (20.77, 25.02)	24.47 (3.49) *	27.16 (23.86, 29.07) *
Smoking status, n (%)						
Never smoking	52 (32.50)	49 (39.52)	52 (35.37) *	177 (99.44)	125 (100.00)	167 (100.00)
Give up smoking	22 (13.75)	20 (16.13)	37 (25.17) *	0 (0.00)	0 (0.00)	0 (0.00)
Smoking now	86 (53.75)	55 (44.35)	58 (39.46) *	1 (0.56)	0 (0.00)	0 (0.00)
Alcohol intake, n (%)						
Never drinking	87 (54.38)	83 (66.94)	65 (44.22)	174 (97.75)	122 (97.60)	163 (97.60)
To give up drinking	24 (15.00)	15 (12.10)	21 (14.28)	0 (0.00)	0 (0.00)	0 (0.00)
Drinking now	49 (30.62)	26 (20.97)	61 (41.50)	4 (2.25)	3 (2.40)	4 (2.40)
Physical activity, n (%)						
Low	50 (31.25)	37 (29.84)	45 (30.61)	39 (21.91)	28 (22.40)	47 (28.14)
Mediate	58 (36.25)	40 (32.26)	52 (35.37)	95 (53.37)	65 (52.00)	86 (51.50)
High	52 (32.50)	47 (37.90)	50 (34.01)	44 (24.72)	32 (25.60)	34 (20.36)
Marital status, n (%)						
Married/cohabiting	143 (89.38)	112 (90.32)	133 (90.48)	151 (84.83)	111 (88.80)	150 (89.82)
Widowed/divorced/separation/single	17 (10.62)	12(9.68)	14 (9.52)	27 (15.17)	14 (11.20)	17 (10.18)
Level of education, n (%)						
Illiteracy	30 (18.75)	16 (12.90)	16 (10.88)	73 (41.01)	44 (35.20)	62 (37.12)
Primary school	41 (25.62)	36 (29.03)	39 (26.53)	52 (29.21)	39 (31.20)	51 (30.54)
Junior secondary and above	89 (55.63)	72 (58.06)	92 (62.59)	53 (29.78)	42 (33.60)	54 (32.34)
Per capita monthly income, n (%)						
<500, RMB	59 (36.87)	55 (44.36)	64 (43.54)	74 (41.57)	49 (39.20)	74 (44.31)
500~, RMB	51 (31.88)	36 (29.03)	38 (25.85)	60 (33.71)	41 (32.80)	49 (29.34)
1000~, RMB and above	50 (31.25)	33 (26.61)	45 (30.61)	44 (24.72)	35 (28.00)	44 (26.35)
Family history of T2DM, n (%)	3 (1.88)	2 (1.61)	11 (7.48) *	1 (0.56)	0 (0.00)	10 (5.99) *
SBP (mmHg)	116.00 (108.00, 127.75)	120.94 (15.31)	124.00 (112.00, 138.00) *	120.50 (111.75, 135.25)	123.00 (114.00, 135.00)	132.00 (120.00, 143.50) *
PP (mmHg)	43.67 (39.67, 51.50)	45.00 (41.33, 51.17)	47.00 (41.67, 54.33) *	50.28 (12.51)	50.60 (12.20)	53.96 (12.83) *
TC (mmol/L)	4.37 (0.71)	4.62 (0.89) *	4.47 (3.99, 5.12)	4.84 (0.77)	4.90 (0.87)	5.01 (1.00)
TG (mmol/L)	1.32 (0.92, 2.04)	1.46 (0.98, 2.25)	1.72 (1.20, 2.94) *	1.43 (1.02, 2.10)	1.84 (1.28, 2.72) *	1.97 (1.42, 2.84) *
HDL−C (mmol/L)	1.29 (1.10, 1.58)	1.24 (1.02, 1.49)	1.15 (0.99, 1.43) *	1.48 (0.36)	1.28 (1.14, 1.55) *	1.30 (0.29) *
LDL−C (mmol/L)	2.65 (0.66)	2.92 (0.80) *	2.61 (0.85)	2.96 (0.69)	2.95 (0.86)	2.89 (2.24, 3.66)
FPG (mmol/L)	4.89 (0.50)	5.21(4.83, 5.56) *	8.25 (7.27, 10.33) *	5.04 (0.46)	5.32 (4.94, 5.81) *	7.96 (7.24, 9.89) *
HbA1c (%)	5.30 (5.10, 5.50)	5.90 (5.70, 6.00) *	7.50 (6.70, 9.10) *	5.40 (5.20, 5.50)	5.90 (5.80, 6.00) *	7.50 (6.70, 9.00) *
INS (pmol/L)	11.64 (9.42, 14.21)	12.30 (9.28, 16.12)	14.11 (10.42, 18.32) *	12.30 (4.38)	12.95 (10.28, 17.28) *	15.79 (12.66, 21.91) *
Progesterone (ng/mL)	0.80 (0.52, 1.05)	0.94 (0.69, 1.36) *	1.30 (0.82, 1.74) *	0.80 (0.56, 1.05)	1.03 (0.72, 1.30) *	1.15 (0.92, 1.64) *
CpG 1 methylation	−4.72 (0.41)	−4.54 (−4.98, −4.24) *	−4.73 (−5.05, −4.43)	−4.68 (−4.97, −4.34)	−4.58 (0.60)	−4.67 (−4.96, −4.35)
CpG 2 methylation	−4.47 (0.31)	−4.44 (−4.66, −4.26)	−4.50 (0.28)	−4.46 (−4.65, −4.21)	−4.43 (0.29)	−4.50 (−4.66, −4.25)
CpG 3 methylation	−4.53 (−4.96, −4.19)	−4.45 (−5.01, −3.98)	−4.49 (−4.92, −4.09)	−4.42 (−4.87, −4.04)	−4.68 (0.85)	−4.52 (0.60)

Values are mean (standard deviation) or median (inter-quartile range) for continuous variables and number (percentages) for categorical variable. The ESRα methylation level in CpG islands (M-value) was calculated as the log2 ratio of the intensities of methylated probe versus unmethylated probe. Abbreviation: BMI, body mass index; CpG, cytosine-phosphoguanine; FPG, fasting plasma glucose; HDL-C, high-density lipoprotein cholesterol; HbA1c, glycosylated hemoglobin A1c; INS, insulin; IFG, impaired fasting glucose; LDL-C, low-density lipoprotein cholesterol; NGT, normal glucose tolerance; PP, pulse pressure; RMB, renminbi; SBP, systolic blood pressure; TC, total cholesterol; TG, triglyceride; T2DM, type 2 diabetes mellitus. * Compared with NGT, *p* < 0.05.

**Table 2:** Associations of *ESRα* methylation (CpG 1) and progesterone with IFG and T2DM.

Variables	Men		Postmenopausal Women	
	Crude Model	Adjusted Model ^a^	Crude Model	Adjusted Model ^a^
**IFG**				
*ESRα* methylation (CpG 1)				
Dichotomies	2.13 (1.32, 3.43) *	1.77 (1.05, 3.00) *	1.70 (1.07, 2.70) *	1.82 (1.09, 3.04) *
T1	Reference	Reference	Reference	Reference
T2	1.10 (0.61, 1.97)	0.99 (0.52, 1.89)	1.09 (0.61, 1.93)	1.23 (0.66, 2.29)
T3	2.42 (1.35, 4.35) *	2.02 (1.06, 3.84) *	1.84 (1.05, 3.24) *	1.98 (1.06, 3.72) *
*P*-trend	0.003	0.030	0.033	0.033
Progesterone				
Dichotomies	2.00 (1.24, 3.22) *	2.03 (1.18, 3.49) *	2.25 (1.41, 3.60) *	2.13 (1.27, 3.56) *
T1	Reference	Reference	Reference	Reference
T2	1.25 (0.70, 2.24)	1.17 (0.62, 2.21)	1.37 (0.76, 2.46)	1.14 (0.61, 2.14)
T3	2.13 (1.19, 3.81) *	2.26 (1.15, 4.47) *	3.19 (1.79, 5.71) *	2.65 (1.42, 4.95) *
*P*-trend	0.011	0.020	<0.001	0.002
**T2DM**				
*ESRα* methylation (CpG 1)				
Dichotomies	1.10 (0.70, 1.71)	1.15 (0.64, 2.05)	1.04 (0.68, 1.58)	1.00 (0.57, 1.73)
T1	Reference	Reference	Reference	Reference
T2	1.02 (0.59, 1.76)	0.83 (0.41, 1.69)	1.37 (0.81, 2.30)	1.42 (0.73, 2.76)
T3	1.29 (0.74, 2.23)	1.56 (0.77, 3.18)	0.97 (0.57, 1.62)	0.89 (0.45, 1.77)
*P*-trend	0.365	0.224	0.895	0.745
Progesterone				
Dichotomies	4.05 (2.52, 6.51) *	3.00 (1.63, 5.52) *	4.45 (2.83, 6.99) *	3.30 (1.85, 5.90) *
T1	Reference	Reference	Reference	Reference
T2	1.65 (0.92, 2.96)	1.52 (0.74, 3.10) *	2.69 (1.55, 4.68) *	2.87 (1.43, 5.77) *
T3	8.29 (4.43, 15.53) *	6.40 (2.83, 14.45) *	6.75 (3.79, 12.03) *	5.28 (2.52, 11.08) *
*P*-trend	<0.001	<0.001	<0.001	<0.001

Abbreviations: *CI*s, confidence intervals; CpG, cytosine-phosphoguanine; *ESRα*, estrogen receptors α; IFG, impaired fasting glucose; *OR*s, odds ratios; T2DM, type 2 diabetes mellitus; T, tertiles. ^a^: adjusted for BMI, smoking status, alcohol intake, physical activity, per capita monthly income, level of education, family history of T2DM, SBP, PP, TC, TG, HDL-C, and LDL-C. * *p* < 0.05.

## Data Availability

No new data were created or analyzed in this study. Data sharing is not applicable to this article.

## Supplementary Materials

The following supporting information can be downloaded at: https://www.mdpi.com/article/10.3390/nu15071659/s1, Table S1. The details of methylation measurement method. Table S2. The primer sequences of *ESRα* gene. Table S3. The *OR*s (95% *CI*) of individual CpG site with IFG and T2DM. Table S4. The *OR*s (95% *CI*) of target genomic regions with IFG and T2DM. Table S5. Associations of *ESRα* methylation (CpG 1) and progesterone levels with IFG and T2DM stratified by alcohol intake in men. Table S6. Associations of *ESRα* methylation (CpG 1) and progesterone levels with IFG and T2DM stratified by smoking status in men. Figure S1. Combined effects of *ESRα* methylation (CpG 1) and progesterone on IFG and T2DM stratified by alcohol intake and smoking status in men.

## The Chinese Clinical Trial Registration

ChiCTR-OOC-15006699 (URL: http://www.chictr.org.cn/showproj.aspx?proj=11375), accessed on 6 July 2015.

## Abbreviations

T2DM	type 2 diabetes mellitus
IFG	impaired fasting glucose
ESRα	estrogen receptor α
IR	insulin resistance
PIK3	phosphatidylinositol-3-kinase
AKT	protein kinase B
GLUT4	glucose transporter 4
CpG	cytosine-phosphoguanine
HOMA	homeostasis model assessment
INS	insulin
HbA1c	glycosylated hemoglobin A1c
NGT	normal glucose tolerance
BMI	body mass index
TC	total cholesterol
TG	triglyceride
HDL-C	high-density lipoprotein cholesterol
LDL-C	low-density lipoprotein cholesterol
FPG	fasting plasma glucose
HPLC	high performance liquid chromatography
LC-MS/MS	liquid chromatography-tandem mass spectrometry
ADA	American Diabetes Association
WHO	World Health Organization
SD	standard deviations
IQR	interquartile ranges
T	tertiles
OR	odds ratios
CI	confidence intervals
E_2_	estradiol
IRS	insulin receptor substrate
