# Supplementary material for: Combined Effects of ESRα DNA Methylation and Progesterone on Glucose Metabolic Disorders: The Henan Rural Cohort Study
Source: Nutrients. 2023 Mar 29;15(7):1659. doi: 10.3390/nu15071659 (PMC10096615; doi:10.3390/nu15071659)
Supplement: Supplementary file 1 [file nutrients-15-01659-s001.zip › Supplement file.pdf]

Legends for supplementary tables and figure

Supplementary Table S1 The details of methylation measurement method.

Supplementary Table S2 The primer sequences of *ESRα* gene.

Supplementary Table S3 The *OR*s (95% *CI*) of individual CpG site with IFG and T2DM.

Supplementary Table S4 The *OR*s (95% *CI*) of target genomic regions with IFG and T2DM.

Supplementary Table S5 Associations of *ESRα* methylation (CpG 1) and progesterone levels with IFG and T2DM stratified by alcohol intake in men.

Supplementary Table S6 Associations of *ESRα* methylation (CpG 1) and progesterone levels with IFG and T2DM stratified by smoking status in men.

Supplementary Figure S1 Combined effects of *ESRα* methylation (CpG 1) and progesterone on IFG and T2DM stratified by alcohol intake and smoking status in men.

Supplementary Table S1 The details of methylation measurement method

	Data	Standard
The mean effective sequencing depth on target	2389X	>1000X
Sample proportions for average effective sequencing depth >500X	99%	>90%
The proportion of sequencing depth >10X among average effective sequencing depth >500X	95%	>90%

20 × 20

Supplementary Table S2 The primer sequences of *ESRα* gene

Chromosome	Start site	End site	Primer sequence<br>Forward	 Reverse	Product Sequence
6	152128479	152128709	GGTAY<br>GGGGT<br>ATATA<br>AGGTA<br>GTATAT<br>TAGAG<br>AAAG	TAAAC<br>ATCAC<br>TCCAA<br>ACACA<br>ACTC	GGCACGGGGCACATAAGGCAGCACATTAGA<br>GAAAGCCGGCCCCTGGATCCGTCTTTCGCGT<br>TTATTTTAAGCCCAGTCTTCCCTGGGCCACCT<br>TTAGCAGATCCTCGTGCGCCCCCGCCCCCTG<br>GCCGTGAAACTCAGCCTCTATCCAGCAGCGA<br>CGACAAGTAAAGTAAAGTTCAGGGAAGCTGC<br>TCTTTGGGATCGCTCCAAATCGAGTTGTGCCT<br>GGAGTGATGTTTA
6	152129066	152128830	TGTGG<br>AGGGT<br>TATGGT<br>TATGG	CRTTC<br>RTCCT<br>AAAAC<br>TACAC<br>TTACT<br>CC	TGTGGAGGGTCATGGTCATGGTCCGTGGCCG<br>CGGGCAGGGTGCAGACCGTGTCCCCGCAGG<br>GCAGAAGGCTCAGAAACCGGCGGGCCACCT<br>GGAAAAAGAGCACAGCCCGAGGTTAGAGGC<br>GACGCAGCGCATGTCCCGCCGACACGCGAG<br>CTCTGGCCCCGGCCCTGCCCCGGGAGCCTGC<br>GGGTCCGGTGAAGCCGGGCGACCCGACGGG<br>AGCAAGTGCAGTCCCAGGACGAACG
6	152129651	152129883	gttYGggg<br>gTTTTG<br>YGTGT<br>AGTT	AAACC<br>TTCCC<br>AAATA<br>ACTCA<br>AAAC	GCCCGGGGGTTCTGCGTGCAGCCCGCGCTGC<br>GTTCAGAGTCAAGTTCTCTCGCCGGGCAGCT<br>GAAAAAAACGTACTCTCCACCCACTTACCGT<br>CCGTGCGAGAGGCAGACCCGAAAGCCCGGG<br>CTTCCTAACAAAACACACGTTGGAAAACCAG<br>ACAAAGCAGCAGTTATTTGTGGGGGAAAACA<br>CCTCCAGGCAAATAAACACGGGGCGCTTTGA<br>GTCACTTGGGAAGGTCT

Chromosome: The chromosome on which the genomic region is located; Start and End site were named as the product's positions on the chromosome; The red "C": CpG site.

20 × 20

Supplementary Table S3 The *OR*s (95%*CI*) of individual CpG site with IFG and T2DM

**Target**	**Chr**	**Position**	**Genome Position**	**Distance to TSS**	**IFG**		**T2DM**	
					***OR*s (95%*CI*)**	***P***	***OR*s (95%*CI*)**	***P***
CpG_1	6	37	152128515	62	1.18 (0.96, 1.45)	0.110077	0.94 (0.75, 1.18)	0.598114
CpG_1	6	50	152128528	75	1.26 (1.00, 1.59)	0.048659	0.80 (0.62, 1.03)	0.077521
CpG_1	6	57	152128535	82	1.32 (1.03, 1.70)	0.028696	0.73 (0.56, 0.96)	0.023898
CpG_1	6	59	152128537	84	1.46 (1.21, 1.77)*	0.000070	1.09 (0.89, 1.33)	0.404154
CpG_1	6	106	152128584	131	1.40 (1.15, 1.71)*	0.000972	1.03 (0.82, 1.28)	0.811353
CpG_1	6	110	152128588	135	1.13 (0.93, 1.37)	0.234786	0.95 (0.76, 1.17)	0.617213
CpG_1	6	116	152128594	141	1.23 (1.01, 1.50)	0.040471	1.00 (0.80, 1.24)	0.984480
CpG_1	6	127	152128605	152	1.22 (1.00, 1.49)	0.051081	0.93 (0.74, 1.16)	0.517479
CpG_1	6	153	152128631	178	1.56 (1.26, 1.92)*	0.000034	1.01 (0.82, 1.25)	0.90211
CpG_1	6	156	152128634	181	1.34 (1.09, 1.64)	0.004959	1.05 (0.85, 1.30)	0.623587
CpG_1	6	197	152128675	222	1.29 (1.05, 1.58)	0.015917	0.90 (0.71, 1.13)	0.341967
CpG_1	6	207	152128685	232	1.37 (1.12, 1.67)	0.002378	1.03 (0.84, 1.26)	0.799360
CpG_2	6	24	152129043	590	0.92 (0.62, 1.38)	0.701481	0.91 (0.57, 1.45)	0.685290
CpG_2	6	30	152129037	584	0.98 (0.69, 1.39)	0.902492	0.74 (0.50, 1.10)	0.136573
CpG_2	6	32	152129035	582	1.28 (0.87, 1.88)	0.207772	0.93 (0.61, 1.42)	0.729509
CpG_2	6	48	152129019	566	1.32 (1.06, 1.65)	0.014983	0.94 (0.74, 1.18)	0.589196
CpG_2	6	56	152129011	558	0.96 (0.65, 1.41)	0.830051	0.87 (0.56, 1.35)	0.531914
CpG_2	6	79	152128988	535	0.96 (0.66, 1.40)	0.824943	0.76 (0.49, 1.17)	0.212922
CpG_2	6	82	152128985	532	0.94 (0.66, 1.33)	0.722450	0.75 (0.49, 1.15)	0.190266
CpG_2	6	109	152128958	505	0.76 (0.54, 1.08)	0.127232	0.76 (0.50, 1.15)	0.189221
CpG_2	6	121	152128946	493	0.87 (0.60, 1.27)	0.466706	0.60 (0.38, 0.92)	0.020766
CpG_2	6	124	152128943	490	0.99 (0.68, 1.45)	0.974153	0.63 (0.42, 0.95)	0.025938
CpG_2	6	129	152128938	485	0.90 (0.55, 1.45)	0.655929	0.58 (0.34, 0.98)	0.043381
CpG_2	6	138	152128929	476	1.03 (0.71, 1.49)	0.877806	0.58 (0.37, 0.92)	0.019231
CpG_2	6	141	152128926	473	1.07 (0.71, 1.60)	0.756399	0.53 (0.33, 0.85)	0.008045
CpG_2	6	146	152128921	468	0.84 (0.57, 1.24)	0.378589	0.64 (0.41, 1.02)	0.060852
CpG_2	6	148	152128919	466	1.00 (0.70, 1.44)	0.989093	0.71 (0.46, 1.12)	0.141502
CpG_2	6	161	152128906	453	0.87 (0.61, 1.25)	0.456161	0.68 (0.45, 1.02)	0.064494
CpG_2	6	172	152128895	442	0.88 (0.61, 1.27)	0.497081	0.68 (0.45, 1.03)	0.067771
CpG_2	6	182	152128885	432	0.98 (0.70, 1.38)	0.903616	0.62 (0.43, 0.90)	0.012933
CpG_2	6	188	152128879	426	1.02 (0.71, 1.47)	0.895525	0.73 (0.49, 1.10)	0.129064
CpG_2	6	197	152128870	417	0.95 (0.67, 1.36)	0.785307	0.72 (0.48, 1.07)	0.104989
CpG_2	6	201	152128866	413	0.84 (0.57, 1.24)	0.389405	0.55 (0.35, 0.84)	0.006342
CpG_2	6	206	152128861	408	0.91 (0.66, 1.26)	0.562304	0.92 (0.62, 1.35)	0.657008
CpG_2	6	209	152128858	405	1.19 (0.85, 1.67)	0.317542	0.73 (0.49, 1.11)	0.138382
CpG_3	6	24	152129674	1221	1.54 (1.09, 2.17)	0.013942	0.66 (0.48, 0.90)	0.008444
CpG_3	6	26	152129676	1223	1.44 (1.03, 2.02)	0.035051	0.82 (0.58, 1.14)	0.232543
CpG_3	6	31	152129681	1228	1.91 (1.37, 2.68)*	0.000166	1.00 (0.74, 1.34)	0.981833
CpG_3	6	51	152129701	1248	1.54 (1.05, 2.25)	0.027165	0.79 (0.57, 1.09)	0.146574
CpG_3	6	54	152129704	1251	1.62 (1.18, 2.24)	0.003205	0.98 (0.73, 1.32)	0.901234
CpG_3	6	71	152129721	1268	1.67 (1.09, 2.55)	0.017569	0.73 (0.51, 1.06)	0.096784
CpG_3	6	91	152129741	1288	1.08 (0.71, 1.63)	0.731084	0.90 (0.64, 1.25)	0.517787
CpG_3	6	95	152129745	1292	1.35 (0.89, 2.05)	0.154121	1.02 (0.73, 1.43)	0.918592
CpG_3	6	99	152129749	1296	1.45 (0.98, 2.14)	0.062628	0.99 (0.73, 1.35)	0.945400
CpG_3	6	112	152129762	1309	1.61 (1.10, 2.34)	0.013907	1.03 (0.75, 1.42)	0.851761
CpG_3	6	120	152129770	1317	1.25 (0.83, 1.89)	0.280955	0.56 (0.36, 0.85)	0.007261
CpG_3	6	141	152129791	1338	1.47 (1.04, 2.08)	0.028231	0.86 (0.64, 1.15)	0.294123

20 × 20

CpG_3	6	204	152129854	1401	1.38 (1.07, 1.78)	0.011766	1.08 (0.86, 1.35)	0.516127
CpG_3	6	209	152129859	1406	0.91 (0.73, 1.13)	0.401470	1.02 (0.82, 1.27)	0.864557

Abbreviation: Chr, chromosome; Distance to TSS, the relative distance (in bp) to transcriptional start site (TSS), and the minus sign indicates that the site is at the upstream of TSS; IFG, impaired fasting glucose; T2DM, type 2 diabetes mellitus; CpG, cytosine-phosphoguanine.

Adjusted for BMI, smoking status, alcohol intake, physical activity, per capita monthly income, level of education, family history of T2DM, SBP, PP, TC, TG, HDL-C, and LDL-C.

**P* <0.05 /49 considered as statistically significant with two-tailed test.

Supplementary Table S4 The ORs (95% CI) of target genomic regions with IFG and T2DM

Genomic region	Length	Distance to TSS	Men		Postmenopausal women	
			IFG	T2DM	IFG	T2DM
Chr6: 152128479_Chr6: 152128709 (CpG 1)	231	26	1.77 (1.04, 3.00)*	1.15 (0.64, 2.05)	1.82 (1.09, 3.04)*	1.00 (0.57, 1.73)
Chr6: 152129066_Chr6: 152128830 (CpG 2)	237	377	1.37 (0.81, 2.32)	0.65 (0.36, 1.18)	1.32 (0.80, 2.17)	0.78 (0.46, 1.33)
Chr6: 152129651_Chr6: 152129883 (CpG 3)	233	1198	1.17 (0.68, 1.99)	0.95 (0.53, 1.69)	0.77 (0.46, 1.30)	1.04 (0.59, 1.84)

Abbreviations: Genomic region, Genomic region was named as the start site to the end site on the chromosome; Length: The product's size(bp); Distance to TSS: The relative distance (in bp) to transcriptional start site (TSS), and the minus sign indicates that the site is at the upstream of TSS; IFG, impaired fasting glucose; T2DM, type 2 diabetes mellitus; CpG, cytosine-phosphoguanine.

Adjusted for BMI, smoking status, alcohol intake, physical activity, per capita monthly income, level of education, family history of T2DM, SBP, PP, TC, TG, HDL-C, and LDL-C.

The methylation levels of CpGs were divided into dichotomous variables based on their corresponding median values.

**P*<0.05.

20 × 20

Supplementary Table S5 Associations of *ESRα* methylation (CpG 1) and progesterone levels with IFG and T2DM stratified by alcohol intake in men

Variables	Adjusted *OR*s (95% *CI*s) [a]	
	**IFG**	**T2DM**
***ESRα* methylation (CpG 1)**		
**No drinking now**		
Dichotomies	2.28 (1.22, 4.24)*	1.48 (0.71, 3.09)
T1	Reference	Reference
T2	1.09 (0.51, 2.29)	1.08 (0.43, 2.69)
T3	2.25 (1.05, 4.82)*	2.55 (1.01, 6.48)*
P-trend	0.035	0.042
**Drinking now**		
Dichotomies	1.06 (0.28, 4.08)	0.65 (0.20, 2.15)
T1	Reference	Reference
T2	2.96 (0.55, 16.02)	0.68 (0.15, 3.08)
T3	3.03 (0.62, 14.87)	0.67 (0.15, 2.88)
P-trend	0.174	0.575
**Progesterone**		
**No drinking now**		
Dichotomies	2.28 (1.23, 4.24)*	3.75 (1.74, 8.08)*
T1	Reference	Reference
T2	1.27 (0.63, 2.56)	1.40 (0.59, 3.35)
T3	2.78 (1.25, 6.17)*	12.27 (4.03, 37.39)*
P-trend	0.015	<0.001
**Drinking now**		
Dichotomies	1.00 (0.24, 4.19)	2.60 (0.70, 9.60)
T1	Reference	Reference
T2	0.43 (0.06, 3.21)	1.46 (0.30, 7.10)
T3	0.90 (0.14, 5.79)	2.80 (0.54, 14.54)
P-trend	0.846	0.193

Abbreviation: *CI*, confidence interval; CpG, cytosine-phosphoguanine; *OR*, odds ratio; IFG, impaired fasting glucose; T2DM, type 2 diabetes mellitus; T, tertiles.

[a]: adjusted for BMI, smoking status, physical activity, per capita monthly income, level of education, family history of T2DM, SBP, PP, TC, TG, HDL-C, and LDL-C.

*P<0.05.

Supplementary Table S6 Associations of *ESRα* methylation (CpG 1) and progesterone levels with IFG and T2DM stratified by smoking status in men

Variables	Adjusted *OR*s (95% *CI*s) [a]	
	IFG	T2DM
***ESRα* methylation (CpG 1)**		
**No smoking now**		
Dichotomies	1.33 (0.62, 2.84)	1.17 (0.50, 2.71)
T1	Reference	Reference
T2	0.45 (0.16, 1.22)	0.96 (0.33, 2.82)
T3	1.28 (0.50, 3.24)	1.43 (0.50, 4.07)
P-trend	0.419	0.474
**Smoking now**		
Dichotomies	1.81 (0.77, 4.26)	1.47 (0.57, 3.76)
T1	Reference	Reference
T2	2.42 (0.85, 6.89)	0.67 (0.22, 2.06)
T3	2.53 (0.90, 7.12)	2.26 (0.72, 7.09)
P-trend	0.073	0.224
**Progesterone**		
**No smoking now**		
Dichotomies	2.50 (1.14, 5.48)*	3.15 (1.35, 7.36)*
T1	Reference	Reference
T2	1.63 (0.68, 3.90)	1.73 (0.66, 4.57)
T3	2.65 (0.96, 7.32)	9.56 (2.66, 34.33)*
P-trend	0.057	0.001
**Smoking now**		
Dichotomies	1.56 (0.65, 3.76)	2.44 (0.88, 6.72)
T1	Reference	Reference
T2	0.77 (0.26, 2.28)	1.13 (0.33, 3.90)
T3	1.57 (0.55, 4.54)	3.38 (0.99, 11.54)
P-trend	0.347	0.042

Abbreviation: *CI*, confidence interval; CpG, cytosine-phosphoguanine; *OR*, odds ratio; IFG, impaired fasting glucose; T2DM, type 2 diabetes mellitus; T, tertiles.

[a]: adjusted for BMI, alcohol intake, physical activity, per capita monthly income, level of education, family history of T2DM, SBP, PP, TC, TG, HDL-C, and LDL-C.

*$P<0.05$.

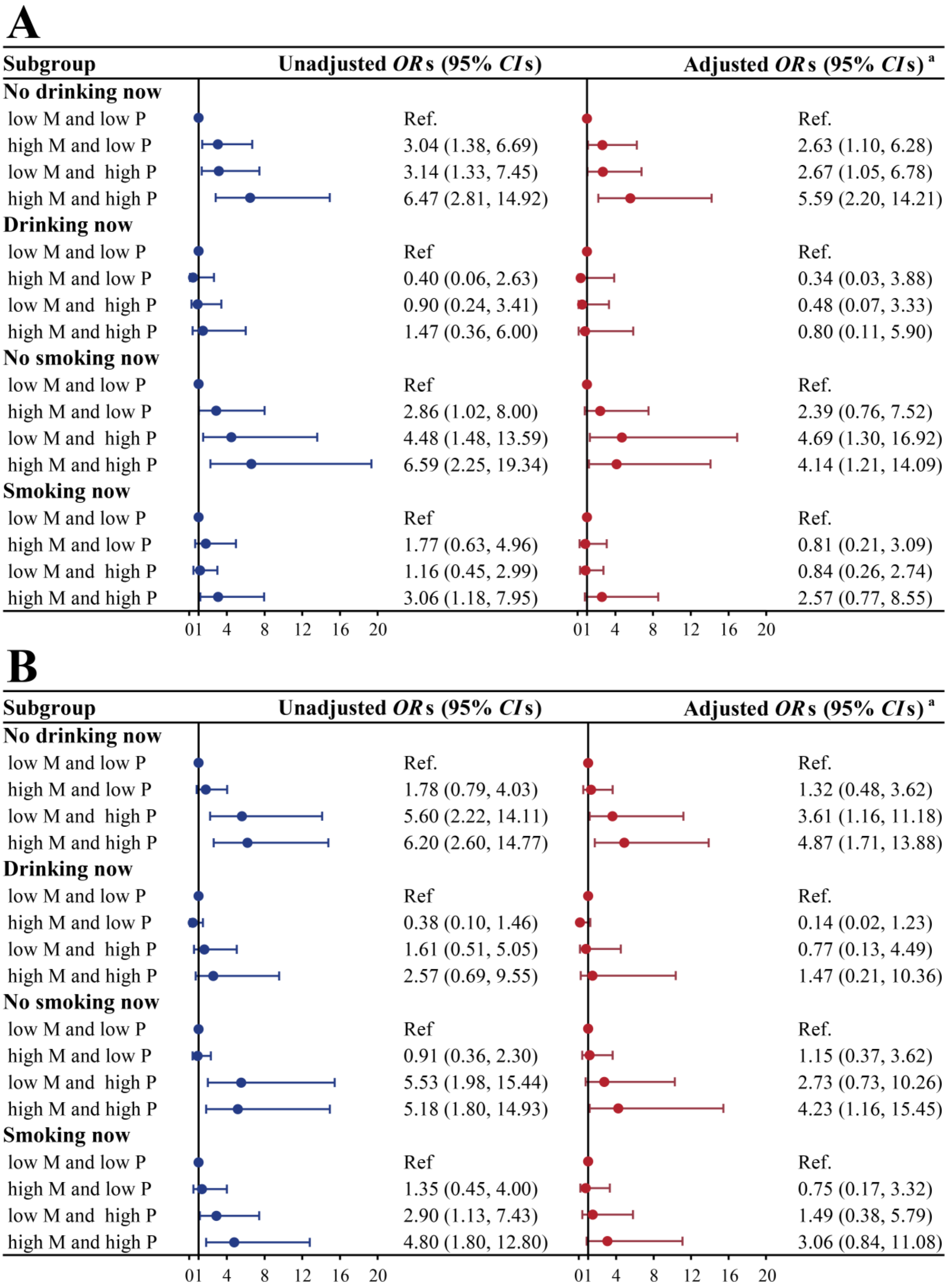

Supplementary Figure S1 Combined effects of *ESRα* methylation (CpG 1) and progesterone on IFG and T2DM stratified by alcohol intake and smoking status in men. (A) presented the combined effect of *ESRα* methylation and progesterone on IFG; (B) presented the combined effect of *ESRα* methylation and progesterone on T2DM. [a]: adjusted for BMI, smoking status, alcohol intake, physical activity, per capita monthly income, level of education, family history of T2DM, SBP, PP, TC, TG, HDL-C, and LDL-C; The

20 × 20

stratification variable was omitted from the model for each stratified analysis. Abbreviations: *CI*, confidence interval; CpG, cytosine-phosphoguanine; *OR*, odds ratio; M, *ESRα* methylation; P, progesterone.
